# Proposed protocol for selection of living kidney donors with diabetes excludes >99% of people with diabetes

**DOI:** 10.1111/ctr.15179

**Published:** 2023-11-13

**Authors:** Mark Gilchrist, Kashyap Patel

**Affiliations:** Institute of Biomedical and Clinical Sciences, University of Exeter Medical School, Exeter, UK

There is an urgent need to increase the living kidney donor pool to allow life-transforming renal transplantation. People with diabetes are generally not considered suitable candidates for kidney donation due to the high future risk of chronic kidney disease (CKD) in these individuals. However, there is a considerable variation in CKD risk in people with diabetes and some will never develop CKD. These individuals could potentially donate a kidney without a significant adverse effect on their personal health and can increase the living donor pool.

In support of this, we welcome the recent Organ Procurement and Transplantation Network (OPTN) policy amendments which proposed the recommendation for consideration of potential donors with diabetes^1^. The guidelines suggest exclusion of individuals with evidence of end organ damage or who have an unacceptable lifetime risk complications^1^. This over-arching guidance is broadly in line with the individualised approach recommended by the British Transplantation Society (BTS)^2^. How these guidelines should be applied in clinical practice is unclear.

No thresholds for measurable risk factors are proposed in the OPTN guidance. In a recent commentary, Soliman and colleagues highlighted the need for clarity on defining thresholds for acceptable risk across multiple relevant risk factors for progression of CKD in diabetes^3^. They propose a set of criteria against which to assess suitability for living kidney donation in people with diabetes. To date this is the only published protocol based on the current OPTN guidance.

However, we believe that these criteria need substantial revision to achieve the aim of permitting meaningful numbers of people with diabetes to proceed safely to donation. We believe these criteria are too restrictive and will effectively remove almost all individuals with diabetes.

To assess how many people with diabetes could be eligible for living kidney donation, we applied these criteria in 28822 people with diabetes from a large population cohort from the UK (n=502538 UK Biobank)^4^. Diabetes was defined as detailed in our previous work ^5^. Participants in receipt of a kidney transplant prior to inclusion in the Biobank were excluded. Median age was 61.5 years (range 40.2 – 72.1 years), median duration of diabetes was 3.7 years (range 0 to 67 years), median eGFR 92 mls/min/1.73m^2^ (range 3 to 160 mls/min/1.73m^2^) Application of the full set of criteria leaves only 18/19869 individuals (0.09%) with diabetes to be considered eligible for donation. Two of these individuals would potentially be excluded due to previous malignancy leaving 16 potentially eligible donors (0.08%, 16/19869), median age 63 year (range 56-69.1 years), median eGFR 96mls/min/1.73m^2^ (range 69-104 mls/min/1.73m^2^).

The UK Biobank is recognised as having a significant healthy volunteer bias with health outcomes and life expectancy being substantially better for participants than the general population of the United Kingdom^5^. HbA1c was measured at a single time point rather than the 3 recommended by Soliman et al’s proposals. We did not account for the number of oral hypoglycaemic agents being taken by participants. All of these are only likely to reduce the proportion of eligible donors in a real world setting further highlighting the restrictive nature of the proposed recommendation.

It is widely recognised that the prevalence of diabetes will continue to rise globally. This brings the double-edged sword of a greater prevalence of ESKD and a diminution of the prospective live donor pool. Much more effort is needed to increase the live donor pool and, in this regard, we support the aims of Organ Procurement and Transplantation Network (OPTN) policy. However, our data suggest that without clear thresholds for what constitutes acceptable risk, most centers interpreting their recommendations will effectively remove all patients with diabetes thus making this recommendation impractical for use in current clinical practice. Further work is needed to identify useful criteria to use in clinical practice. It is possible that we will never be able to identify a large pool of potential donors with diabetes with similar lifetime risk for advanced CKD as those without diabetes. However, the analysis of recently available large scale diabetes cohorts and registry data with longitudinal Electronic Healthcare Records (such as the Million Veteran Programme, Geisinger MYCODE and the UK Clinical Practice Research Datalink) can provide an exciting starting point to identify the appropriate criteria.

## Figures and Tables

**Table 1:** Impact of each criteria proposed by Soliman et al on numbers remaining eligible after exclusion of those who are outside recommended parameters.

Criteria from Soliman et al (n with data)	Number eligible
Total with Diabetes at baseline	28822
CKD-Epi eGFR >60 (27480)	24553
Age >55 (28822)	21849
Non-insulin-dependent (23727)	22229
BMI <30 (28550)	13061
DM duration >3years (28822)	14077
Non smoker (28511)	12443
No hypertension (28822)	10554
Normal fundus exam & no evidence of other end-organ complications (28822)	26049
Controlled hyperlipidemia as per ADA guidelines (21061)	3963
Urine ACR <30mg/g or urine PCR <200 mg/g (25939)	22111
HbA1c <7% o≥ three occasions in past 2 years. (28822)	15418
Eligible if all categories are applied (19869)	18
